# Quantitative evaluation of the spinal cord compression in patients with cervical spondylotic myelopathy using synthetic MRI

**DOI:** 10.3389/fphys.2023.1140870

**Published:** 2023-04-10

**Authors:** Qiufeng Liu, Haoyue Shao, Chaoxu Liu, Weiyin Vivian Liu, Azzam Saeed, Qiya Zhang, Jun Lu, Guiling Zhang, Li Li, Xiangyu Tang, Guanghui Du, Wenzhen Zhu

**Affiliations:** ^1^ Department of Radiology, Tongji Hospital Affiliated to Tongji Medical College, Huazhong University of Science and Technology, Wuhan, China; ^2^ Department of Orthopedics, Tongji Hospital Affiliated to Tongji Medical College, Huazhong University of Science and Technology, Wuhan, China; ^3^ MR Research, GE Healthcare, Beijing, China; ^4^ Department of Urology, Tongji Hospital Affiliated to Tongji Medical College, Huazhong University of Science and Technology, Wuhan, China

**Keywords:** magnetic resonance image, synthetic, quantitative evaluation, spinal cord, cervical spondylotic myelopathy

## Abstract

**Objectives:** This work aimed to investigate the feasibility and diagnostic value of synthetic MRI, including T1, T2 and PD values in determining the severity of cervical spondylotic myelopathy (CSM).

**Methods:** All subjects (51 CSM patients and 9 healthy controls) underwent synthetic MRI scan on a 3.0T GE MR scanner. The cervical canal stenosis degree of subjects was graded 0—III based on the method of a MRI grading system. Regions of interest (ROIs) were manually drawn at the maximal compression level (MCL) by covering the whole spinal cord to generate T1_MCL_, T2_MCL_, and PD_MCL_ values in grade I-III groups. Besides, anteroposterior (AP) and transverse (Trans) diameters of the spinal cord at MCL were measured in grade II and grade III groups, and relative values were calculated as follows: rAP = AP_MCL_/AP_normal_, rTrans = Trans_MCL_/Trans_normal_. rMIN = rAP/rTrans.

**Results:** T1_MCL_ value showed a decreasing trend with severity of grades (from grade 0 to grade II, *p* < 0.05), while it increased dramatically at grade III. T2_MCL_ value showed no significant difference among grade groups (from grade 0 to grade II), while it increased dramatically at grade III compared to grade II (*p* < 0.05). PD_MCL_ value showed no statistical difference among all grade groups. rMIN of grade III was significantly lower than that of grade II (*p* < 0.05). T2_MCL_ value was negatively correlated with rMIN, whereas positively correlated with rTrans.

**Conclusion:** Synthetic MRI can provide not only multiple contrast images but also quantitative mapping, which is showed promisingly to be a reliable and efficient method in the quantitative diagnosis of CSM.

## 1 Introduction

Cervical spondylotic myelopathy (CSM) is a chronic compressive spinal cord lesion (Rao, 2002; McCormick et al., 2003). It is the most common form of spinal cord injury in adults, especially in older patients (McCormick et al., 2020). It is important to identify early symptoms and provide effective treatments before developing irreversible spinal cord damage (Edwards et al., 2003).

Magnetic resonance image (MRI) is widely used for CSM diagnosis *via* visualization of the anatomical extent of spinal cord compression and the intramedullary signal changes within the spinal cord (Takahashi et al., 1987; Al-Mefty et al., 1988; Ramanauskas et al., 1989; Mehalic et al., 1990; Batzdorf and Flannigan, 1991; Ohshio et al., 1993; Shabani et al., 2019). Conventional MRI, which usually includes T1-and T2-weighted images (T1WI and T2WI), can provide high-resolution images of vertebrae, spinal cord, and surrounding soft tissues (Harkins et al., 2016). However, alterations of the T1 and T2 signal intensity still limit the diagnosis of early stages of CSM (Karpova et al., 2010). A sensitive and reproducible imaging technique is needed for early diagnosis and quantification of spinal cord compression. Quantitative MRI might be an option because that the T1 mapping had shown the clinical potential (Maier et al., 2019; Maier et al., 2020), while T2 and proton density (PD) mappings have been rarely reported.

Synthetic MRI can provide quantitative mapping including T1, T2 and PD mapping as well as multiple contrast-weighted imaging such as T1-, T2-weighted images, simultaneously (Warntjes et al., 2008). Synthetic MRI technique has been widely used in many regions and has shown good diagnostic performance in brain, skeleton, articulatio, breast, prostate and lumbar intervertebral disk degeneration (Hagiwara et al., 2017; Cui et al., 2020; Liu et al., 2021; Moran, 2021; Zhao et al., 2021). To our knowledge, there is no application of synthetic MRI on CSM patients. Therefore, our study aimed to explore the diagnostic value of the quantitative mapping generated by synthetic MRI in evaluating spinal cord compression in patients with CSM.

## 2 Materials and methods

### 2.1 Subjects

The institutional review board of research ethics approved this study and all subjects gave written informed consent. From August 2021 to March 2022, a total of 60 subjects (26 men and 34 women) were involved in this study including 51 patients with CSM and 9 healthy controls. The 51 patients were diagnosed with CSM by the standard of narrowed anteroposterior diameter of spinal canal revealed by sagittal and axial T2WI MRI, and all the CSM patients in our study were in acute stage with clinical signs of myelopathy. According to the MRI grading system proposed by Kang et al. (2011), all subjects were classified into four grades: 1) grade 0: no spinal canal stenosis is visible (healthy controls); 2) grade I: the spinal canal is narrowed without compression of the spinal cord; 3) grade II: the spinal cord is deformed; 4) grade III: the spinal cord is compressed with additional increased signal intensity near the compressed level on T2 weighted images. Exclusion criteria for subjects were as follows: 1) regular contraindications for MRI, such as claustrophobia and metal implants; 2) patients with prior neurologic trauma or coexisting neurologic disorders; 3) patients with prior surgery. Clinical scores of patients and controls were evaluated using the Japanese Orthopaedic Association (JOA) score. The JOA ranged from 0 to 17 points and correspondingly reflected severe deficits to normal function.

### 2.2 MRI protocol

All subjects underwent MR exams in the supine position on a 3.0 T whole-body scanner (SIGNA Architect, GE Healthcare, Milwaukee, United States) using a 19 channel high-resolution head and neck coil. All subjects were instructed not to move and swallow during scanning to minimize motion artifacts. Routine sequences were acquired, including Sagittal T2WI FSE (TR:2000 ms, TE:102.0 ms, NEX:1.00, Thickness:3.0, Spacing: 0.5), Sagittal FS (Fat Saturation) T2WI FSE (TR:2816 ms, TE:102.0 ms, NEX:2.00, Thickness:3.0, Spacing: 0.5), Sagittal T1WI FSE (TR:612 ms, TE:10.0 ms, NEX:2.00, Thickness:3.0, Spacing: 0.5). Synthetic MRI (MAGiC, MAGnetic resonance image Compilation) scan of the spinal cord was performed at 0.5 mm in-plane resolution and 4 mm slice thickness in multiple axial sections perpendicular to the spinal cord. Other imaging parameters for MAGiC were as follows: TR: 4008 ms, TE: 29.3 ms, spacing: 1.0mm, Matrix size: 400 × 400, NEX: 1.00, scanning time: 7min45s.

### 2.3 Image analysis

**FIGURE 1:**
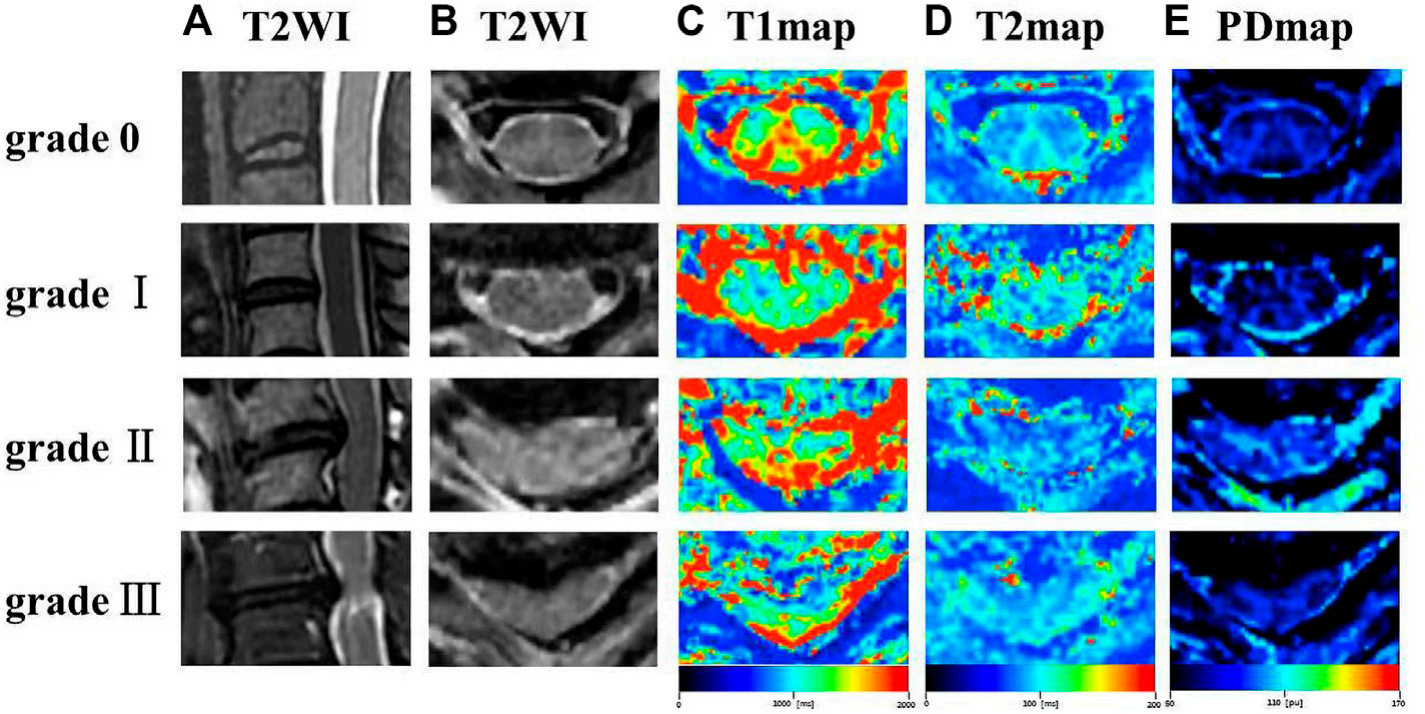
Representation sagittal T2WI **(A)**, axial T2WI **(B)**, T1 colormap **(C)**, T2 colormap **(D)** and PD colormap **(E)** of the spinal cord of at C2/3 intervertebral disc level in grade 0 group, and the spinal cord at the maximal compression level (MCL) in grade I-III groups.

**FIGURE 2:**
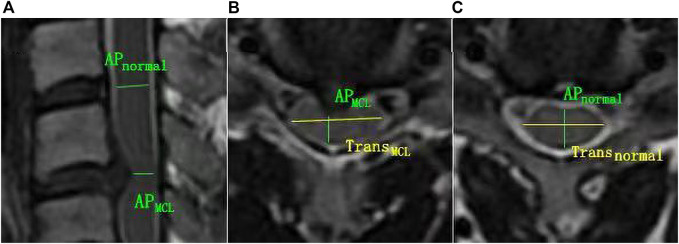
The schematic diagram for diameter measurement. **(A, B)** AP_MCL_ = anteroposterior diameter of the spinal cord at MCL, and Trans_MCL_ = transverse diameter of the spin cord at MCL. **(A, C)** AP_normal_ = anteroposterior diameter of normal level spin cord nearest the MCL. Trans_normal_ = transverse diameter of normal level spin cord nearest the MCL.

The T1, T2, PD maps were generated by an offline post-processing software (SyMRI 11.2.2; SyntheticMR, Linköping, Sweden) in addition to automatically generated multiple contrast images, including T1WI, T2WI, PDWI simultaneously. Two radiologists with ten and 15 years of experience in spine diagnosis respectively evaluated the grade of spinal canal stenosis (SCS) and measured T1, T2 and PD values for all participants. Regions of interest (ROIs) were manually drawn at the maximal compression level (MCL) on synthetic images by covering the whole spinal cord to generate T1_MCL_, T2_MCL_, and PD_MCL_ values in grade I-III groups, and the MAGiC quantitative values of grade 0 group were defined as the average values of spinal cord at C2/3-C6/7 intervertebral disc levels (Figure 1). Besides, the anteroposterior (AP) and transverse (Trans) diameters of the spinal cord on axial imaging were measured in grade II and III groups. The select sections of the spinal cord for measurement were the MCL and the normal level nearest the MCL, as shown in Figure 2. Relative values of AP and Trans diameters were calculated as follows: rAP = AP_MCL_/AP_normal_, rTrans = Trans_MCL_/Trans_normal_. The compression ratio was defined as following: rMIN = rAP/rTrans.

### 2.4 Statistical analysis

All data were analyzed using SPSS 23.0 software (IBM, Armonk, NY) and shown as mean ± standard deviation (SD). The values of T1, T2, PD and clinical score were compared between different CSM grades using Mann-Whitney U test. The correlations among the clinical score, MAGiC quantitative values (T1, T2 and PD), and the diameter values (rAP, rTrans and rMIN) in grade II - III groups were assessed by the Spearman correlation. Intraclass correlation coefficient (ICC) was calculated for the inter-observer consistency of T1, T2 and PD measurements and defined as poor (<0.40), fair (0.40–0.59), good (0.60–0.74), and excellent (0.75–1.00). *p*-values below 0.05 were considered statistically significant.

## 3 Results

### 3.1 Patient characteristic

The mean age and SD of 51 patients with CSM and 9 healthy controls was respectively 47.6 ± 14.1 years and 32.3 ± 12.5 years. Twenty-three (45%) patients were male [4 (44%) male controls]. There were 9 subjects with grade 0, 25 subjects with grade I of CSM, 18 subjects with grade II and 8 subjects with grade III. Demographics of the participants are shown in Table 1. The maximum extension of the CSM was most frequently found in the C5/C6 segment (32/51patients) followed by the C4/C5 segment (10/51patients), C3/4 (6/51patients) and C6/C7 segment (3/51patients). Four of these patients had both a grade II stenosis at C5/C6 and a grade III stenosis at C4/C5. In this case, the statistical analysis only involved the grade III stenosis.

**TABLE 1:** Demographics of healthy controls and patients with cervical spondylotic myelopathy (CSM).

Variables	
Total, n	60
Sex (male/female)	27/33
Healthy controls, n (male/female)	9 (4 men/5 women)
Patients with CSM, n (male/female)	51 (23 men/28 women)
Grade, n	Grade 0,9
	Grade I, 25
	Grade II, 18
	Grade III, 8
Grade, Age (years)	Grade 0,32.3 ± 12.5
	Grade I, 45.1 ± 14.2
	Grade II, 49.9 ± 14.8
	Grade III, 50.0 ± 12.3
Mean age of Grade I-III (years)	47.6 ± 14.1

### 3.2 Synthetic MRI parameters

Mean and standard deviation of all MAGiC quantitative values of the spinal cord at MCL were measured and recorded (Table 2). ICCs for PD, T1 and T2 ranged from 0.950 to 0.998 and showed good inter-observer consistency.

**TABLE 2:** The mean and standard deviation of MAGiC quantitative values (T1, T2 and PD), clinical score, and diameters values of the spinal cord at MCL in different grades of CSM patients.

	Grade 0	Grade I	Grade II	Grade III
T1_MCL_ (ms)	1,309.5 ± 143.0	1,198.9 ± 290.1	1,072.2 ± 114.9	1,491.7 ± 145.2
T2_MCL_ (ms)	87.3 ± 4.0	89.1 ± 5.6	86.7 ± 4.4	91.5 ± 3.6
PD_MCL_ (ms)	79.8 ± 7.8	77.9 ± 6.8	77.0 ± 8.0	80.8 ± 7.2
JOA	16.3 ± 0.9	16.1 ± 1.2	13.1 ± 1.8	11.3 ± 1.6
rAP	-	-	0.884 ± 0.067	0.763 ± 0.134
rTrans	-	-	1.179 ± 0.126	1.264 ± 0.111
rMIN	-	-	0.780 ± 0.133	0.644 ± 0.090

MAGiC, MAGnetic resonance image Compilation; MCL, maximal compression level; PD, proton density; CSM, cervical spondylotic myelopathy; JOA, japanese orthopaedic association; AP, anteroposterior; Trans, transverse; rMIN, rAP/rTrans.

**FIGURE 3:**
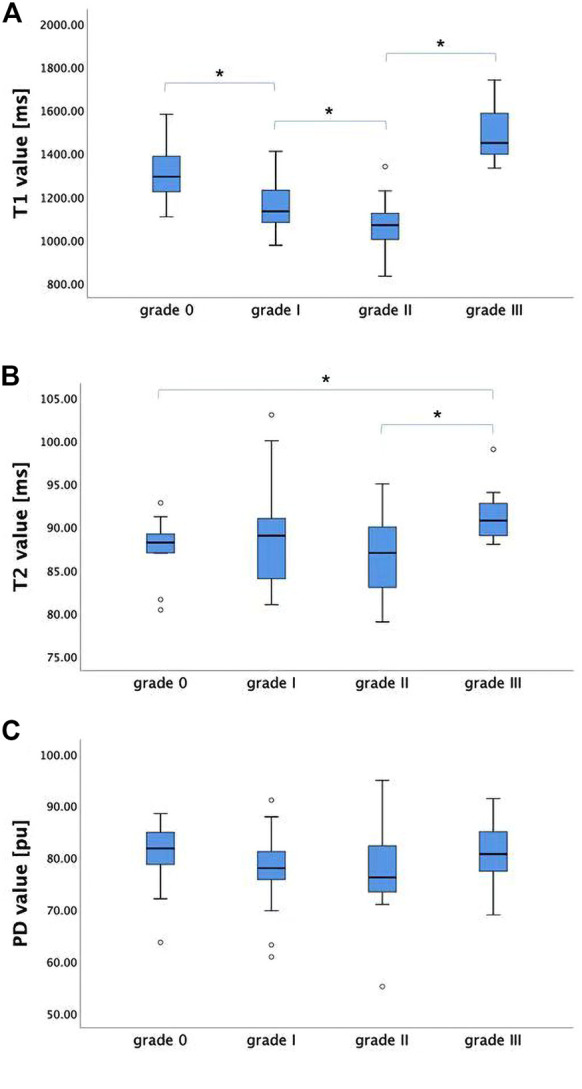
The T1, T2, and PD values (mean ± standard deviation) of grade 0-III groups. **(A)** T1_MCL_ value showed a decreasing trend with severity of grades (from grade 0 to grade II), while it increased dramatically at grade III. **(B)** T2_MCL_ value showed no significant difference among grade groups (from grade 0 to grade II), while it increased dramatically at grade III compared to grade II. **(C)** PD_MCL_ value showed no statistically difference among all grade groups. * Statistically significant at a threshold of *p* < 0.05.

For the maximal compression level, T1 value showed a decreasing trend with severity of grades (from grade 0 to grade II, *p* < 0.05), while it increased dramatically at grade III. Significant differences were found between adjacent groups from grade 0 to grade III. T2_MCL_ value showed no significant difference among grades (from grade 0 to grade II), while it increased dramatically at grade III (*p* < 0.05). PD_MCL_ value showed no statistically difference among all grades. The results were shown in Figure 3.

### 3.3 Association between clinical score, MAGiC quantitative values (T1, T2 and PD), and diameter values of the spinal cord at MCL

**FIGURE 4:**
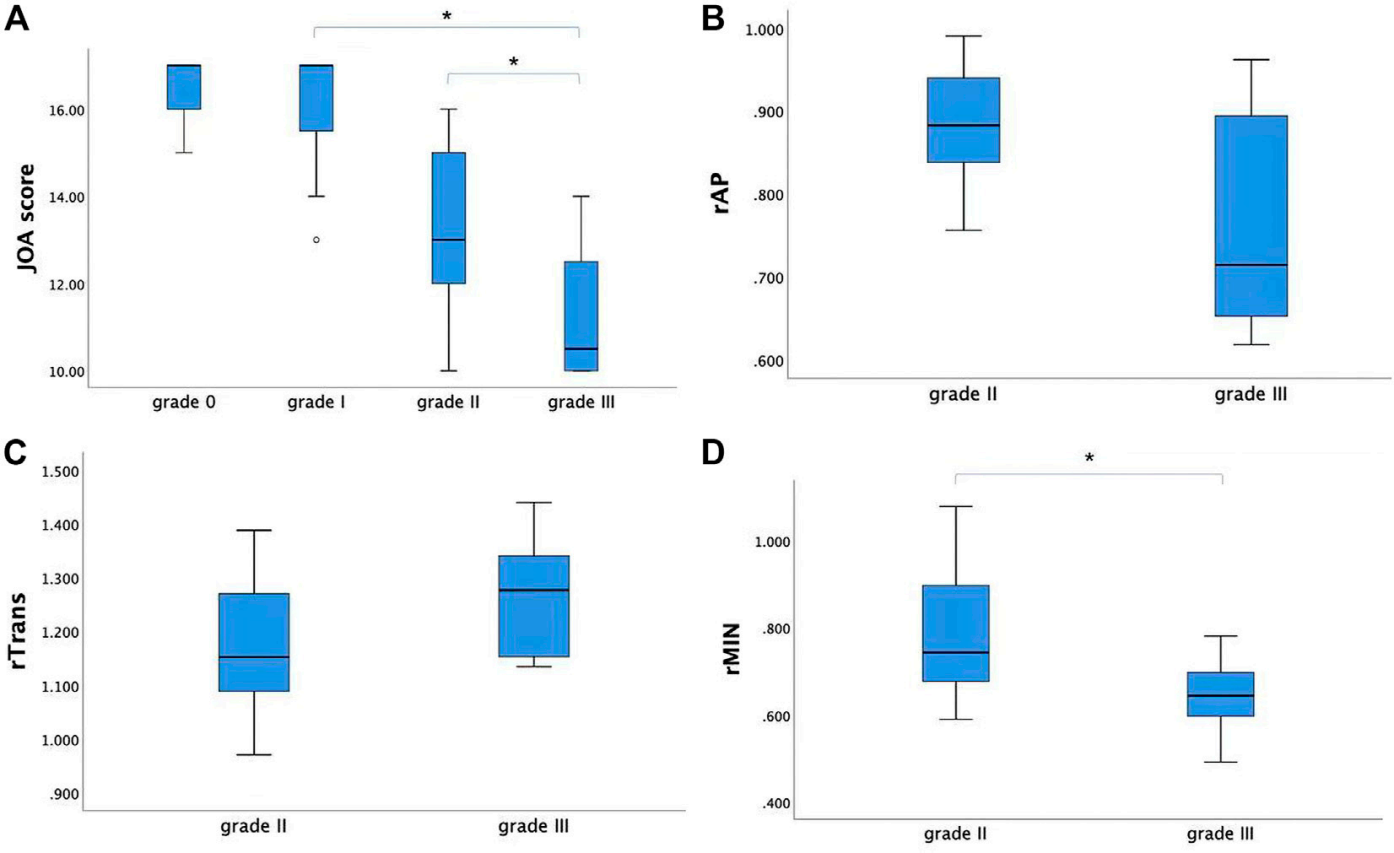
**(A)** The JOA score showed a decreasing trend with severity of grades (from grade I to grade III). **(B, C)** For the CSM patients in grade II and grade III, rAP and rTrans of the spinal cord at MCL showed no significant difference between two groups. **(D)** While rMIN of grade III was significantly lower than that of grade II. * Statistically significant at a threshold of *p* < 0.05.

**FIGURE 5:**
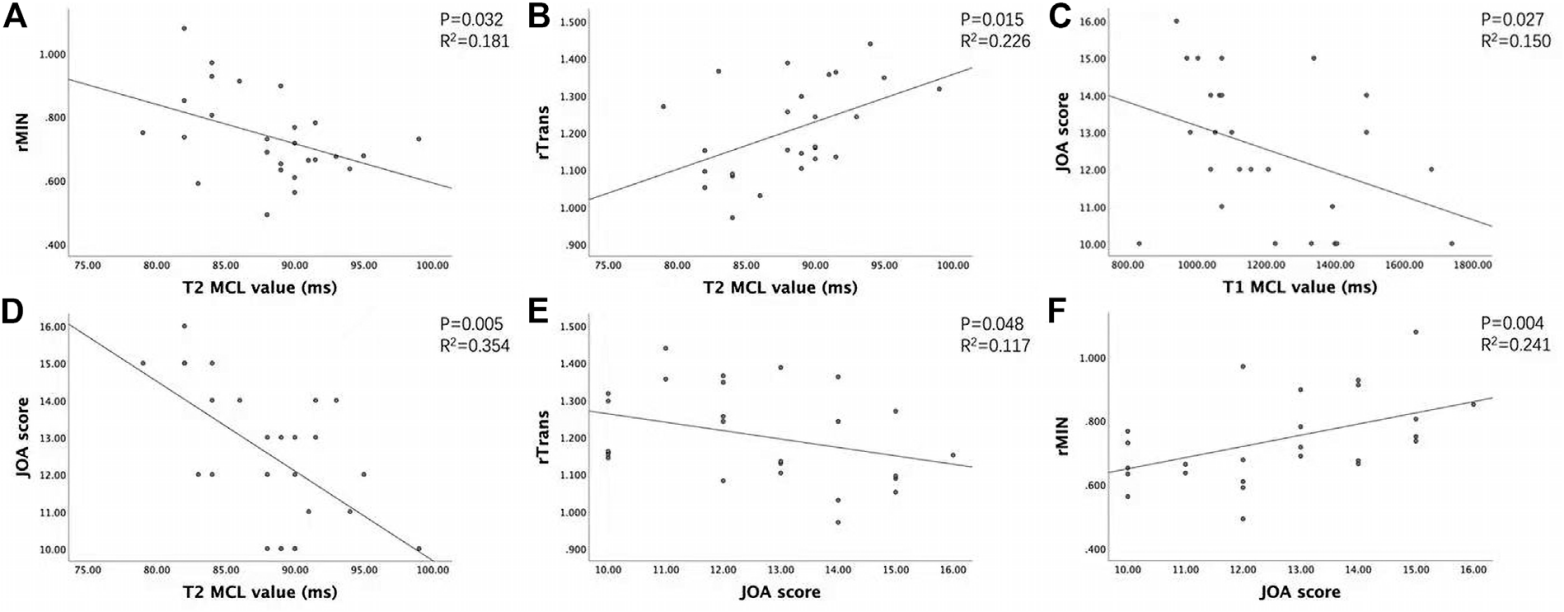
Association between clinical score, MAGIC quantitative values (T1, T2), and diameter values of the spinal cord at MCL in grade II and grade III groups. **(A)** T2_MCL_ value was negatively correlated with rMIN. **(B)** T2_MCL_ value was positively correlated with rTrans. **(C, D)** T1_MCL_ and T2_MCL_ value all negatively correlated with JOA scores. **(E, F)** JOA score negatively correlated with rTrans, whereas positively correlated with rMIN.

The JOA score showed a decreasing trend with severity of grades (from grade I to grade III, *p* < 0.05). For the CSM patients in grade II and grade III, rAP and rTrans of the spinal cord at MCL showed no significant difference between two groups, while rMIN of grade III was significantly lower than that of grade II (*p* < 0.05), as shown in Figure 4. Multilevel correlations were observed in the CSM patients of grade II and III. T1_MCL_ and T2_MCL_ values were all negatively correlated with JOA scores. T2_MCL_ value was negatively correlated with rMIN, whereas positively correlated with rTrans. Conversely, JOA score was negatively correlated with rTrans, whereas positively correlated with rMIN (Figure 5).

## 4 Discussion

The current study was the first to use synthetic MRI in the diagnosis of CSM patients. Multiple relaxation maps (T1, T2 and PD maps) as well as contrast-weighted images were obtained in a single scan of synthetic MRI. Our study demonstrated that T1 and T2 relaxation time of spinal cord at MCL changed with a grade dependent difference and T1_MCL_ value could sensitively reflect the microstructural change of compressive spinal cord even at grade I stage. Moreover, T1 and T2 values were related to clinical score and diameters values of the spinal cord at MCL.

In our study, T1, T2 and PD maps as well as multi-contrast images were generated from a one-time-scan of MAGiC imaging sequence. The acquisition time (7 min 45 s) of MAGiC sequence is obviously shorter than that of the conventional mapping sequence, which is nearly 20 minutes for only T1 mapping (Maier et al., 2019; Maier et al., 2020). Therefore, it reduces patient’s discomfort, motion artifacts and increases work efficiency, allowing to easily apply in clinics. Overall, the range of T1 values in our study was consistent with that in a previous study (Maier et al., 2019) using T1 mapping in diagnosis of CSM, and T2 value obtained from MAGIC and T2 mapping data exhibited strong positive correlation in Jiang’s study (Jiang et al., 2020), so the MAGiC quantitative data should be practicable. In our result, T1_MCL_ showed a decreasing trend with the severity of grades (from grade 0 to grade II, *p* < 0.05), which is in accordance with Maier’s study (Maier et al., 2019). Further, a reduced myelin water fraction was found in CSM patients, suggesting that a reduction in myelin water is a surrogate for spinal cord compression and dysfunction (Liu et al., 2017). Therefore, the main contribution to T1 decrease should be a direct physical consequence of compression compacting the microstructure and squeezing free water molecules with long T1 relaxation times out of the affected spinal cord. Nevertheless, T1 value decreased significantly in grade I group, even though the spinal cord did not present with deformation on sagittal T2-weighted MRI, which was explained by a kinetic MRI technique (Zeng et al., 2016) showing that intermittent compression and deformation of the spinal cord takes place in grade I patients. However, T1_MCL_ and T2_MCL_ increased dramatically together at grade III, which has not appeared in previous studies, one may speculate that tissue edema, inflammation, gliosis and microstructural damage occurred at the site of maximum compression (Batzdorf and Flannigan, 1991, Meyer et al., 2008). In addition, T2_MCL_ value showed no significant difference among grades from grade 0 to grade II, and PD_MCL_ value showed no statistical difference among all grades, indicating that T1_MCL_ value could more sensitively reflect the microstructural change of compressive spinal cord than T2_MCL_ and PD_MCL._ Meanwhile, the result means that T2_MCL_ increase is associated with unfavorable clinical outcomes and poor responses to decompressive surgery (Sampath et al., 2000; Meyer et al., 2008), and microstructural damages of spinal cord in grade I and grade II may be reversible.

To explain the dramatic change of T1_MCL_ and T2_MCL_ values from grade II to grade III, we compared the diameter values of the spinal cord at MCL in grade II and grade III groups and tried to find the association of clinical score, MAGiC quantitative values, and diameter values of the spinal cord at MCL. Our study demonstrated that T2_MCL_ value was negatively correlated with rMIN, besides, rMIN of grade III was significantly lower than that of grade II (*p* < 0.05), but rAP and rTrans of the spinal cord at MCL showed no significant difference between two groups. The result indicated that the T2 values ascended only when the spinal cord endured an oblate compression rather than local compression. In that case, the function of the compressed spinal cord would have less chance to be compensated by surrounding tissues, which could explain the result that JOA score positively correlated with rMIN and negatively correlated with rTrans, whereas no correlation with rAP in our study. Meanwhile, our study demonstrated that T1_MCL_ and T2_MCL_ value all negatively correlated with JOA scores in grade II and grade III patients, which is inconsistent with a previous T1 mapping study showing no correlation between T1 relaxation times and clinical signs of CSM (Maier et al., 2020). A likely explanation is the fact that we only compared the relationship between T1_MCL_, T2_MCL_ value and JOA in grade II and grade III patients, and all the CSM patients in our study were in acute stage.

There were some limitations in this study. Firstly, the sample size in this study was relatively small, especially the amount of CSM patients in grade II and III groups. Secondly, there was no electrophysiological study to diagnose central conduction deficits in our study. However, all the CSM patients we enrolled were in acute stage to provide a relative objective clinical score. Third, the post-operation patients will also be included to explore the feasibility of synthetic MRI for predicting postoperative outcomes.

## 5 Conclusion

In this study, Synthetic MRI is showed promisingly to be a reliable and efficient method in the quantitative diagnosis of CSM. Synthetic MRI can provide not only multiple contrast images but also quantitative mapping. The MAGiC quantitative values are correlated with clinical signs and diameter changes at MCL. Therefore, Synthetic MRI may open up new dimensions in providing quantitative measures in assessing CSM and eventually improve the diagnosis of CSM in the future.

## Data Availability

The raw data supporting the conclusions of this article will be made available by the authors, without undue reservation.

## Abbreviations

CSM, cervical spondylotic myelopathy; MCL, maximal compression level; MAGiC, MAGnetic resonance image Compilation; PD, proton density; ROIs, Regions of interest; JOA, Japanese Orthopaedic Association; ICC, Intraclass correlation coefficient; SCS, spinal canal stenosis; AP, anteroposterior; Trans, transverse.
